# Prediction of Driver’s Intention of Lane Change by Augmenting Sensor Information Using Machine Learning Techniques

**DOI:** 10.3390/s17061350

**Published:** 2017-06-10

**Authors:** Il-Hwan Kim, Jae-Hwan Bong, Jooyoung Park, Shinsuk Park

**Affiliations:** 1 Hyundai Motor Company, Hwaseong-si 18280, Korea; kihwan2@hyundai.com; 2 Department of Mechanical Engineering, Korea University, Seoul 02841, Korea; delitian@korea.ac.kr; 3 Department of Control and Instrumentation Engineering, Korea University, Sejong 30019, Korea

**Keywords:** advanced driver assistance system (ADAS), lane change, driver’s intention, artificial neural network (ANN), support vector machine (SVM)

## Abstract

Driver assistance systems have become a major safety feature of modern passenger vehicles. The advanced driver assistance system (ADAS) is one of the active safety systems to improve the vehicle control performance and, thus, the safety of the driver and the passengers. To use the ADAS for lane change control, rapid and correct detection of the driver’s intention is essential. This study proposes a novel preprocessing algorithm for the ADAS to improve the accuracy in classifying the driver’s intention for lane change by augmenting basic measurements from conventional on-board sensors. The information on the vehicle states and the road surface condition is augmented by using an artificial neural network (ANN) models, and the augmented information is fed to a support vector machine (SVM) to detect the driver’s intention with high accuracy. The feasibility of the developed algorithm was tested through driving simulator experiments. The results show that the classification accuracy for the driver’s intention can be improved by providing an SVM model with sufficient driving information augmented by using ANN models of vehicle dynamics.

## 1. Introduction

As the number of vehicles increases worldwide, the traffic situation becomes increasingly complicated in terms of safety. The automotive industry has been developing various safety technologies, and driver assistance systems, such as headway distance control, automatic braking system and evasive steering system, have become one of the major features of a vehicle for the safety of the driver and passengers. As an active safety system, the advanced driver assistance system (ADAS) has been developed to assist the driver for improved safety and better vehicle control. The ADAS equipped with advanced sensors and intelligent video systems is designed to alert the driver to potential traffic hazards or to take over control of the vehicle to avoid impending collisions and accidents. The ADAS is activated when the predetermined conditions for the driver’s operation and the state of the vehicle are met. In conventional ADAS, a threshold is set for driver’s control input, such as the steering wheel angle, the steering wheel angular velocity, or the pedal position. If the driver’s control input is greater than the predetermined threshold, the ADAS is activated. In the activation of ADAS, however, there can be conflicting situations where the intervention of the ADAS can interfere with the driver’s intention of operation. Correct prediction of driver’s intention is an essential part to determine whether the ADAS should engage to override the driver’s control inputs [1].

For the most time during driving, the driver is required to maneuver the steering wheel, and the lane change maneuver is one of the main causes of road traffic accidents [2]. It was reported that the percentage of fatal accidents related to lane change increased from 18% in 2005 to 23.6% in 2014, while the total number of fatal crash in the U.S. gradually decreased from 50,000 to 38,000 during the same period [3]. ADAS technologies, such as Lane Support Systems, Lane Keeping Assistance System (LKAS) and Lane Departure Warning System (LDWS), enable automated lane control. The lane change control of the ADAS is based on the driver’s control input and surrounding traffic situation. With the current technologies of the ADAS, however, there are possibilities of unwanted lane change against the driver’s intention, which may lead to a situation that endangers the safety of the driver’s vehicle and its surrounding vehicles.

To alleviate the risk of misjudging the driver’s intention, many studies have attempted to incorporate machine learning techniques to identify the driver’s intention for lane change control with the ADAS [4,5,6,7,8,9,10]. Machine learning has proven its utility in estimation, classification and prediction of system behaviors. For identification of the driver’s intention, in particular, many researchers have investigated classification techniques, such as Hidden Markov Model (HMM), Support Vector Machine (SVM), and Bayesian network. Kuge et al. (2000) [4] developed an HMM-based steering behavior model for emergency lane change, normal lane change, and lane keeping. They reported that the classification accuracy of the model was higher than 98.3%. Jin et al. (2011) [5] developed an algorithm for lane change recognition using the steering wheel angle and the angular velocity as input data to a HMM model. With their method, the classification accuracies for lane change left (LCL), lane change right (LCR) and lane keeping (LK) were 84%, 88% and 94%, respectively. Tran et al. (2015) [6] investigated the performance of a HMM-based system with two different sets of inputs: one only with the driver’s control input (steering wheel angle and gas and brake pedal positions) and the other both with the driver’s control input and with the vehicle states (velocity, acceleration and yaw rate). It was confirmed that with the driver’s control input and the vehicle states the HMM model shows far superior performance in terms of classification time and accuracy. Mandalia and Salvucci (2005) [7] compared by experiment the overlapping window method with the non-overlapping window method. The accuracy of the overlapping method was about 1.2% higher than the non-overlapping method. Aoude et al. (2011) [8] compared SVM- and HMM-based methods in classification of law-abiding and violating drivers. They reported that the SVM-based method has higher accuracies that the HMM-based method in most cases. Kumar et al. (2013) [9] proposed a machine learning algorithm that combines SVM and Bayesian filter. Relevance vector machine (RVM) is an SVM-based Bayesian inference model for probabilistic classification. Morris and Doshi (2011) [10] introduced a RVM model that is capable of classifying the driver’s intention within 3 s before an actual lane change happens. Liu et al. (2010) [11] employed the parallel Bayesian network (PBN) to identify the driver’s lane change behavior. They reported that the PBN model can reduce the response time and error rate. Schubert et al. (2010) [12] developed a classification technique for lane change maneuvers by using camera vision and a radar sensor. In other studies, the Bayesian network was employed to classify the driver intention [13,14,15,16,17].

To classify the driver’s intention at a high level of accuracy, abundant information on the vehicle states should be provided to the machine learning algorithms. The studies mentioned above employed rather expensive sensors to measure various vehicle states, such as the lateral velocity, the heading angle, the side slip angle and the lateral position, to identify the driver’s intention for lane change. Those sensors, however, are impractical to be used in commercial passenger vehicles. Recently, many commercial vehicles are being equipped with on-board sensors to provide basic measurements, such as the steering wheel angle, the yaw rate, the longitudinal and lateral accelerations, and the wheel speed, at an affordable cost [18]. While the on-board sensors are unable to provide the ADAS algorithm with sufficient information on the vehicle states, the vehicle states other than the direct measurements from the on-board sensors may be estimated using machine learning techniques based on the measured data. Along with the vehicle states, the road condition such as the friction coefficient of the road surface is an important factor to be considered to classify the driver’s intention for lane change, and it can also be estimated from the vehicle states measured by the on-board sensors [19,20].

While complex vehicle dynamics models with nonlinear differential equations can be used in augmenting the information on the vehicle states and the road surface condition, real-time computation of numerical integration requires great computation power [21]. For fast real-time computation, purely numerical models with better computational efficiency, such as artificial neural network (ANN), were suggested rather than physical mathematical models of vehicle dynamics [22,23]. An ANN model of vehicle dynamics is suitable for real-time information augmentation, since it only requires summation and product operations of matrices, rather than time-consuming numerical integration of nonlinear differential equations.

In this study, we propose a novel preprocessing algorithm as a practical solution to improving the accuracy of the ADAS in determining the driver’s intention for lane change by augmenting basic measurements from conventional on-board sensors. The inputs to the algorithm include the measured data from the on-board sensors and the augmented vehicle states along with the road surface condition estimated from the measured data. The vehicle states and the road surface condition are estimated by using ANN models that simulate nonlinear dynamics of the vehicle and the interaction between the tires and the road. The ANN models trained by the data obtained from a driving simulator provide augmented information on the vehicle states and the road condition based on the limited information from the on-board sensors. The augmented information from the ANN models along with the direct sensor measurements is then fed to an SVM mode to classify the driver’s intention for lane change. The effectiveness of the proposed algorithm was verified through driving simulator experiments, and the experimental results show that the classification accuracy for the driver’s intention can be improved by providing an SVM model with sufficient driving information augmented by using ANN models of vehicle dynamics and vehicle-road interaction.

This paper is organized as follows: Section 2 illustrates the preprocessing algorithm for the ADAS developed in this study. Section 3 describes the driving simulator experiments to evaluate the performance for the proposed algorithm, and Section 4 presents the experimental results. Finally, Section 5 contains conclusions and future work directions. 

## 2. Classification of Driver’s Intention for Lane Change Using Augmented Sensor Information

**Figure 1:**
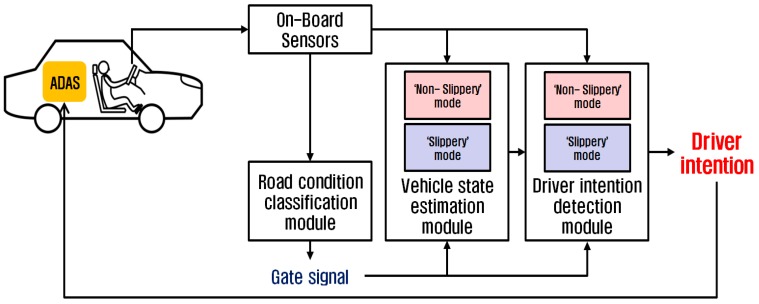
A schematic diagram of the system developed for driver intention classification.

**Figure 2:**
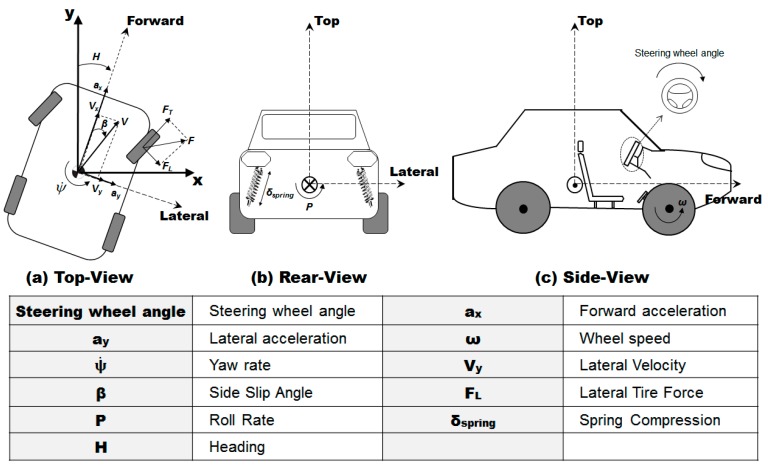
Vehicle states measured by on-board sensors and estimated by ANN models.

This section describes the preprocessing algorithm for the ADAS, which detects the driver’s intention for lane change based on the augmented information on the road surface condition and the vehicle states. Figure 1 illustrates the schematic diagram of the algorithm. The augmented information is estimated from the basic measurements acquired from the on-board sensors commonly equipped on commercial passenger vehicles. The on-board sensors provide the basic measurements of the vehicle states and the driver’s control inputs: the measured vehicle states include the longitudinal and lateral accelerations, the yaw rate, and the wheel speed, while the measured driver’s control inputs include the steering wheel angle and the throttle position. Based on the sensor measurements, the algorithm estimates the road surface condition (non-slippery or slippery). The estimated condition of the road surface, along with the sensor measurements, is then used to estimate the vehicle states that cannot be measured by the on-board sensors. The vehicle states augmented by estimation include the lateral velocity, the side slip angle, the lateral tire force, the roll rate, the suspension spring compression, and the heading direction (Figure 2). The augmented information on the vehicle states is then provided to the algorithm to classify the driver’s intention for lane change. The identified driver’s intention is used to determine whether to activate the ADAS and override the driver’s control inputs for lane change.

The preprocessing algorithm for the ADAS consists of three main modules as illustrated in Figure 1: the *road condition classification* module, the *vehicle state estimation* module and the *driver intention detection* module. The road condition classification module determines whether the road surface condition is non-slippery or slippery by using an ANN-based pattern recognition technique. The vehicle state estimation module augments the vehicle states by using an ANN model representation of vehicle dynamics. The driver intention detection module identifies the driver’s intention for lane change by using an SVM model with the augmented information as inputs.

### 2.1. ANN Models for Road Condition Classification and Vehicle State Estimation

ANN-based models are used for the road condition classification module and the vehicle state estimation module of the preprocessing algorithm for the ADAS. Artificial neural network (ANN) is a computational learning approach inspired by how biological neural networks learn from experiences. Since ANN can effectively solve nonlinear problems of vehicle dynamics, an ANN model can be a pertinent solution to augmenting sensor information that is insufficient to determine the driver’s intention for lane change.

**Figure 3:**
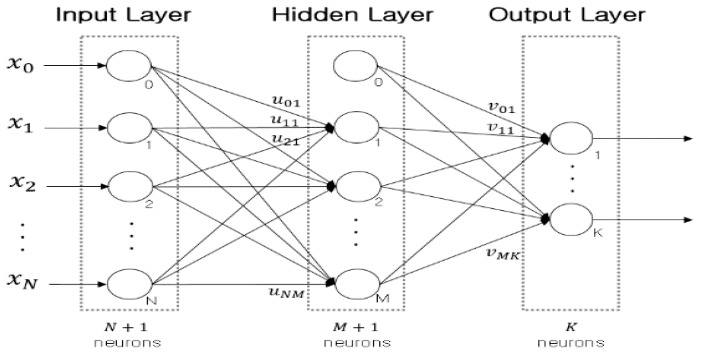
Basic network architecture of three-layered ANN.

The basic structure of the three-layered ANN is illustrated in Figure 3, where the network consists of an input layer, a hidden layer, and an output layer. Each node in the hidden layer and the output layer has an activation function, which defines the output of that node given its own input. The type of the activation function can be chosen properly based on the purpose of the network. In learning phase, the weighted connections between nodes of the network are adjusted.

#### 2.1.1. Road Condition Classification Module

Table 1 summarizes the friction coefficients of four road conditions: dry asphalt, gravel, wet, and snowy [24,25]. In this study, dry asphalt and gavel are grouped as the non-slippery road condition, and wet and snowy are grouped as the slippery road condition. The road condition classification module classifies the road surface conditions into the two classes: non-slippery and slippery.

The road condition classification module is designed to activate when the throttle signal is detected, since the identification of the road friction coefficient is easier during acceleration or deceleration than during constant-speed driving. The signals from the on-board sensors from the time the driver step one’s foot on the acceleration pedal to the time when the driver takes one’s foot off the pedal are used to determine the road condition.

**Figure 4:**
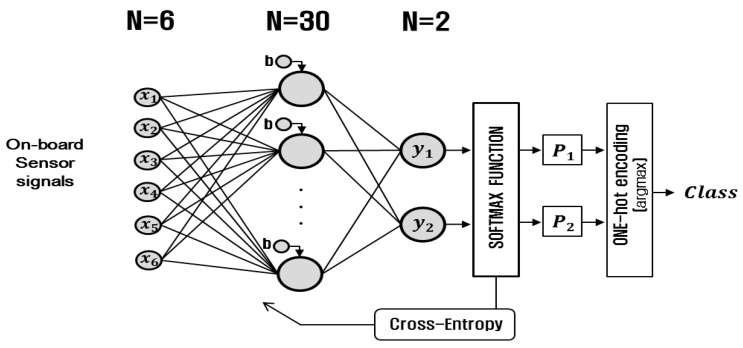
Artificial neural network model for road condition classification module.

The road condition classification module has the structure of the three-layered ANN with the softmax activation function in the output layer. The three-layered model was employed based on the guideline suggested by Panchal et al. (2011) [26]. The input, hidden, and output layers have six, thirty, and two nodes, respectively. At the output node, the softmax activation function yields the probability values of the classification represented by the node. For the nodes in the hidden layer, the bipolar sigmoid is commonly used as the activation function. In the training phase of the module, the performance index defined by cross-entropy is minimized, and in the testing phase, the class labels are determined by applying the one-hot-encoding to the output probability values. The detailed architecture of the neural network is shown in Figure 4. In the input layer, the six nodes are for the signals from the on-board sensors (the longitudinal and lateral accelerations, the yaw rate, the wheel speed, the steering wheel angle and the throttle position). 

#### 2.1.2. Vehicle State Estimation Module

The vehicle state estimation module estimates the vehicle states based on the data from the on-board sensors and the road condition classification module. This module uses the NARX (nonlinear autoregressive with exogenous input) neural network, which is a type of recurrent neural network particularly useful for time series analysis. 

The NARX model is known to be an effective tool for time series prediction compared with other feedforward ANN models, since it enables to relate the current value of a time series to the past values of the time series and the exogenous inputs [27,28].

The mathematical form of the NARX model is given as follows:

(1) $$\hat{y}\left(k\right)=f\left[y\left(k-1\right),\;y\left(k-2\right);\;u_{1}\left(k-1\right),\;u_{1}\left(k-2\right),\;u_{1}\left(k-3\right);\;u_{2}\left(k-1\right),\;u_{2}\left(k-2\right),\;u_{2}\left(k-3\right);\;\ldots \;;\;u_{5}\left(k-1\right),\;u_{5}\left(k-2\right),\;u_{5}\left(k-3\right)\right]$$

where u(k)∈ℝ and $\hat{y}\left(k\right)\in \mathbb{R}$ denote the inputs and output of the model at discrete time step *k*, respectively. The filter orders for input u(k) and output $\hat{y}\left(k\right)$ are $d_{u}$ = 3 and $d_{y}=2$, respectively. f(·) is a nonlinear function with universal approximation capability. The nonlinear function f(·) of the NARX model plays an important role to model nonlinear relations among the vehicle states of vehicle dynamics.

There are two modes for the NARX model: series-parallel (SP) mode and parallel (P) mode. SP mode is mainly used for single step prediction or short term prediction since the values from the previous step are inserted as an input vector for the prediction at the next step. P mode has a feedback loop structure and the estimated output values are included as an input vector of network, and its performance is better than SP mode in multi-step or mid-and-long term prediction tasks [29,30,31]. Combinations of the two modes can be used for training and testing of the neural network [31,32,33,34,35]. For the vehicle state estimation module, P mode was used for training and testing of the module, since the module is designed to carry out long term prediction.

**Figure 5:**
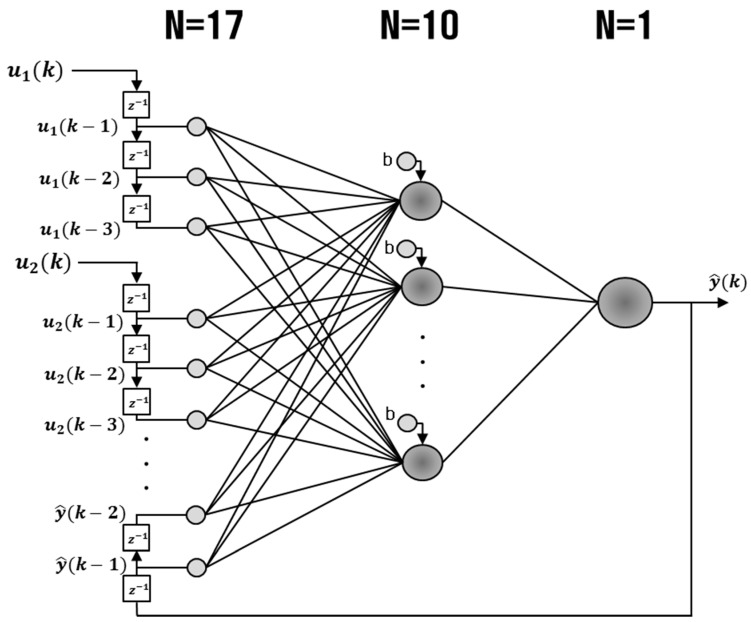
NARX Neural network model for estimation of vehicle state parameters ($z^{-1}$ is the unit time delay).

The structure of the NARX neural network in P mode is shown in Figure 5. The NARX neural network has a multi input-single out (MISO) structure. To yield six vehicle states (the lateral velocity, the side slip angle, the lateral tire force, the roll rate, the suspension spring compression, and the heading direction), six separate NARX models are required. Each NARX model consists of the input layer, the output layer, and the hidden layer. The input, hidden and output layers have 17, 10 and one nodes, respectively. In the hidden layer, the bipolar sigmoid function is used as the activation function. For the output layer, a linear activation function is employed for the activation function. Five measurements from the on-board sensors (the longitudinal and lateral accelerations, the yaw rate, the wheel speed, and the steering wheel angle) and the estimated output are used as inputs to the nodes in the input layer. For each of the five measurements from the on-board sensors, the values at two time steps and one time step before the present time and the present time are used ($d_{u}$ = 3). For the estimated input, the estimation at one step before the present time and the presentation are used ($d_{y}$ = 2). These form the total of 17 inputs. For the hidden layer, 10 nodes were used after having tested the number of nodes from five to 20 nodes. The output layer has one node that yields one of the six vehicle states.

### 2.2. Driver Intention Detection Module

**Figure 6:**
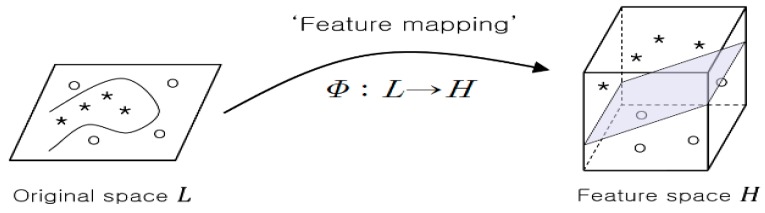
Use of feature map for non-separable problem.

The driver intention detection module classifies the driver’s intention for lane change based on the augmented information from the on-board sensors and the vehicle state estimation module. This module employs a support vector machine (SVM) model for classification. SVM classification is known to have good generalization abilities. For example, binary classification using SVM can find the optimal hyper-plane that maximizes the separation margin between two classes. When dealing with non-separable data, SVM utilizes the feature map *φ* to transform a low dimensional input space into a feature space of a higher dimension where linear classification is more feasible as illustrated in Figure 6.

The problem of finding the optimal hyper plane can be formulated as the constrained optimization problem by introducing the slack variable *ξ* of the so-called soft-margin method, as follows:

(2) $$\mathrm{Minimize}\;J\left(\omega ,\;\xi \right)=\frac{1}{2}\|\omega {\|}^{2}+C\overset{N}{\underset{i=1}{\sum }}\xi_{i}\;\mathrm{Subject}\;\mathrm{to}\;t_{i}\left(\omega^{T}\Phi \left(x_{i}\right)+b\right)\geq 1-\xi_{i}\;,\;i=1,\;\ldots ,\;N\;\xi_{i}\geq 0\;,\;i=1,\;\ldots ,\;N$$

By introducing the Lagrange multipliers and applying the Karush–Kuhn–Tucker (KKT) conditions, Equation (7) becomes the following Lagrange dual problem:

(3) $$\mathrm{Maximize}\;\tilde{L(}\alpha )=\overset{N}{\underset{i=1}{\sum }}\alpha_{i}-\frac{1}{2}\overset{N}{\underset{i=1}{\sum }}\overset{N}{\underset{j=1}{\sum }}\alpha_{i}\alpha_{j}t_{i}t_{j}K\left(x_{i},x_{j}\right)\;\mathrm{Subject}\;\mathrm{to}\overset{N}{\underset{i=1}{\sum }}\alpha_{i}t_{i}=0,\;0\leq \alpha_{i}\leq C,\;i=1,\;\ldots ,\;N$$

where $K\left(x_{i},x_{j}\right)$ is the kernel function defined by the inner-product of two feature vectors *φ*($x_{i}$) and *φ*($x_{j}$). The dual problem of Equation (8) can be solved by utilizing the KKT condition to yield the optimal values for $\alpha_{i}$ and b. With the acquired constants $\alpha_{i}$ and b, the decision function is defined as follows:

(4) $$f\left(x\right)=sgn\left(\sum_{i=1}^{N}\alpha_{i}y_{i}K\left(x,x_{i}\right)+b\right)$$

With the input x, the decision function yields the binary value, either positive or negative, to classify two classes. This can be extended to multi-class classification, such as one-against-all classification.

**Figure 7:**
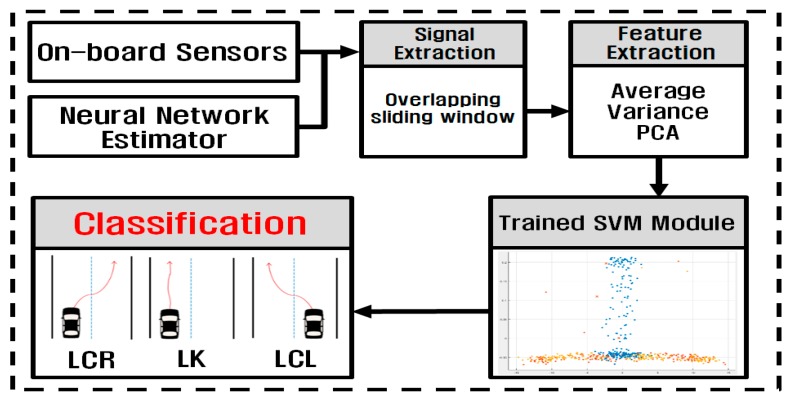
Operation procedure of driver intention recognition using SVM.

Figure 7 shows the schematic diagram of the driver intention detection module. The 11 inputs to the driver intention detection module includes five measurements from the on-board sensors and six vehicle states from the vehicle states estimation module. For signal extraction, overlapping sliding windows are applied to the 11 input signals. It was reported that the overlapping sliding window has better classification ability than the non-overlapping sliding window [7]. The window size and the window slide size used in the module are 0.5 s and 0.2 s, respectively.

For feature extraction, the average and the variance of the windowed signals are calculated, and the principal component analysis (PCA) is performed on the windowed signals. The feature sets obtained from feature extraction are fed to the pre-trained SVM to classify the driver’s intention for lane change into three classes: lane change left (LCL), lane change right (LCR), and lane keeping (LK).

This is a multi-class classification problem with three classes. One-against-all method is employed for the multi-class SVM, and K-fold cross validation is used for the performance evaluation of the driver intention detection module, which is known to be an effective method to deal with a small set of data. For the kernels for training, the quadratic and Gaussian kernels are used for non-slippery and slippery road conditions, respectively.

## 3. Driving Simulation Experiments

**Figure 8:**
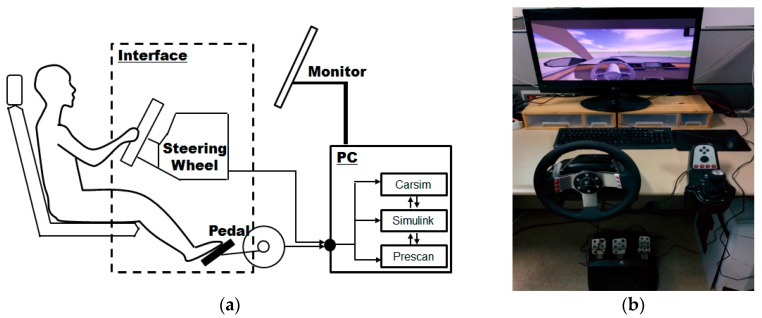
Setup for driving simulator experiments: (**a**) schematic diagram of driving simulator; and (**b**) setup of Steering wheel and pedal for obtaining driving data.

The preprocessing algorithm for the ADAS described in Section 2 was implemented in a PC-based driving simulator (Figure 8). The driving simulator is controlled by PreScan software ver. 7.3 (TASS International, Rijswijk, The Netherlands), CarSim software (Mechanical Simulation Corporation, Ann Arbor, MI, USA), and Simulink (MathWorks, Natick, MA, USA). PreScan is used as a physics-based simulation platform, and CarSim is used for simulation of vehicle dynamics. The vehicle used in the simulator was front-wheel drive. The data from the driving simulator can be collected at the rate of 500 Hz, which is the maximum sampling frequency with PreScan and CarSim software.

A human subject (27-year-old male with six years of driving experience) was instructed to perform three maneuvers of lane change control: lane change left (LCL), lane change right (LCR) and lane keeping (LK). The subject was asked to drive within the speed range 30–80 km/h on a one-way road with three lanes of the width of 3.5 m. The lanes were separated by cones placed at the interval of 50 m.

The three modules of the algorithm were trained using the data collected from the driving simulator. The driving simulator was also used to test the performance of the trained modules.

### 3.1. Training of Road Condition Classification Module

To train the ANN model of the road condition classification module, the driving simulation was performed under four different road surface conditions (dry asphalt, gavel, wet, and snowy) as listed in Table 1. The four road conditions are grouped into two classes (non-slippery for dry asphalt and gavel; slippery for wet and snowy). The module was trained by using a total of 120,000 data sets with 60,000 for the non-slippery road condition and 60,000 for the slippery road condition.

### 3.2. Training of Vehicle State Estimation Module

The vehicle state estimation module was trained under two different road conditions (non-slippery and slippery). For each road condition, six NARX models yield six vehicle states. Thus, the vehicle state estimation module is composed of 12 sub-modules. 

To train each sub-module, the input data from the on-board sensors (five measurements) and the target data from the driving simulator (six vehicle states) were provided to train the vehicle state estimation module by using the Levenberg–Marquart back-propagation algorithm. It should be noted that all 12 sub-modules have the same structure and training data sets, while the target data sets are all different.

For the training of the module, we used 80,000 data sets with 40,000 for the non-slippery road condition and 40,000 for the slippery road condition, which was sampled at 500 Hz. For the two sub-modules to estimate the heading direction in the non-slippery and slippery road conditions, the on-board sensor data, down-sampled at 50 Hz, were used for training, and the total number of the training data sets was 3000 (1500 for the non-slippery road and 1500 for the slippery road).

### 3.3. Training of Driver Intention Detection Module

The driver intention detection module was trained using the input data from the on-board sensors and the vehicle state estimation module and the target data of the driver’s intention of lane change obtained from the questionnaire with the human subject performing the driving simulation. From the windowed signals, the features (the average, the variance, and the principal components) of the signals were extracted to be used as the input to the SVM module. The SVM module was trained to classify the three intentions of the driver labeled as LCL, LCR and LK by using 581 data sets for the non-slippery road and 550 data sets for the slippery road.

The classification performance of the SVM can be improved by selecting the optimal combination of the input signals, rather than using all the available input data [36,37]. We tested six combinations of input signals to the SVM, as listed in Table 2, and compared the classification abilities of the combinations. In the table, the vehicle states estimated by ANN are marked by boldface.

## 4. Experimental Results 

The performance of the three of the three modules of the preprocessing algorithm of the ADAS was tested through driving simulation experiments. The three modules were tested with the driving simulation data that is different from the data used to train the modules. This section presents the experimental results of the road condition classification, the vehicle state estimation and the driver intention detection modules.

### 4.1. Classification of Road Condition 

**Figure 9:**
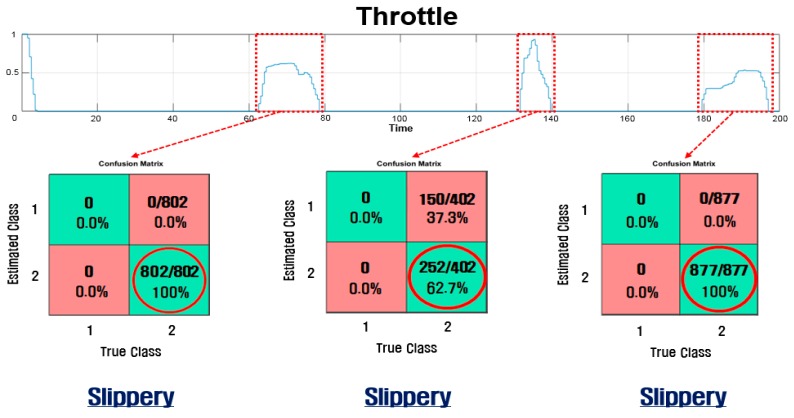
Classification of the road condition while the throttle is on.

The road condition classification determines the road surface based on the on-board sensor signals during the time when the throttle is on. Figure 9 shows the throttle position and the road surface condition estimated while the throttle is on. As shown in the Figure 9, the module correctly classified the road condition (slippery) with a high level of confidence (100%, 62.7%, and 100%). Table 3 lists the results from 52 test trials. Out of 52 trials, there were 51 correct classifications with only one misclassification (highlighted in grey in the Table 3). By using the measurements from the on-board sensors, the module can identify the road surface condition with fairly high accuracy of 98%.

### 4.2. Estimation of Vehicle State Parameters 

**Figure 10:**
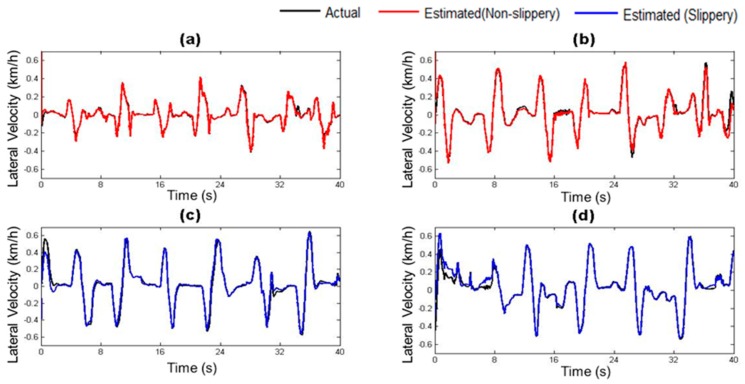
Estimated Lateral Velocity depending on road surface condition: (**a**) Dry asphalt; (**b**) Gravel; (**c**) Wet; and (**d**) Snow.

**Figure 11:**
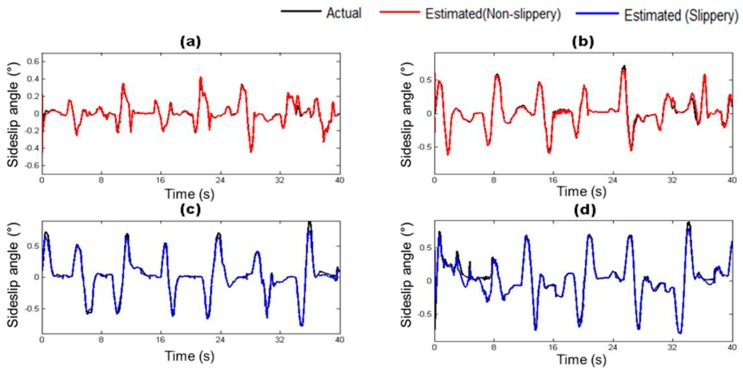
Estimated Side slip angle depending on road surface condition: (**a**) Dry asphalt; (**b**) Gravel; (**c**) Wet; and (**d**) Snowy.

**Figure 12:**
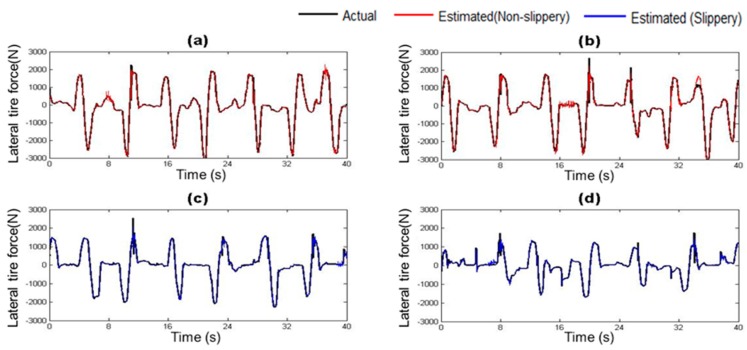
Estimated Lateral Tire Force depending on road surface condition: (**a**) Dry asphalt; (**b**) Gravel; (**c**) Wet; and (**d**) Snowy.

**Figure 13:**
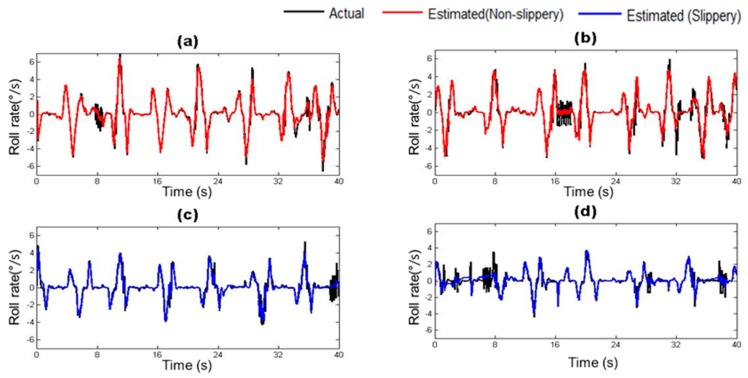
Estimated Roll rate depending on road surface condition: (**a**) Dry asphalt; (**b**) Gravel; (**c**) Wet; and (**d**) Snowy.

**Figure 14:**
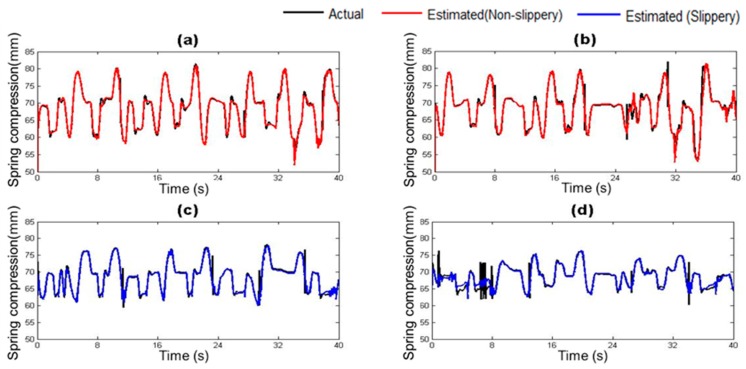
Estimated Suspension Spring Compression depending on road surface condition: (**a**) Dry asphalt; (**b**) Gravel; (**c**) Wet; and (**d**) Snowy.

**Figure 15:**
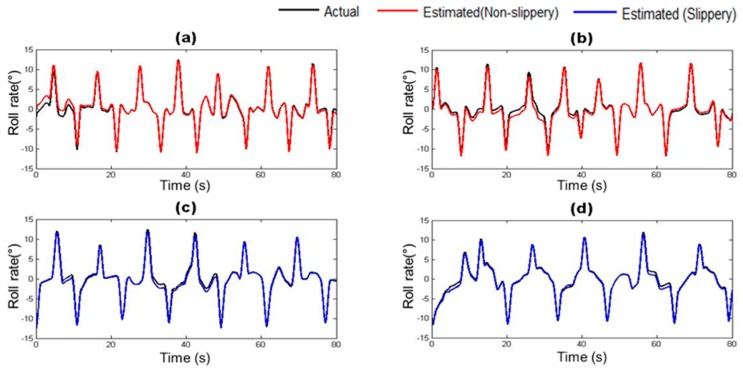
Estimated Heading (Yaw) depending on road surface condition: (**a**) Dry asphalt; (**b**) Gravel; (**c**) Wet; and (**d**) Snowy.

The vehicle states estimated by the trained vehicle state estimation module are compared with those from the on-board sensors of the driving simulator under four different road surface conditions in Figure 10, Figure 11, Figure 12, Figure 13, Figure 14 and Figure 15. As shown in Figure 10, Figure 11, Figure 12, Figure 13, Figure 14 and Figure 15, the estimated lateral velocity, side slip angle, tire lateral force, roll rate, suspension spring compression and heading direction represented by dotted lines are close to those from the on-board sensors of the driving simulator represented by solid lines. 

The errors between the estimated vehicle states and the measured vehicle states were evaluated by the root mean square error (RMSE) and the normalized mean square error (NMSE) given as follows [38]:

(5) $$\mathrm{RMSE}=\sqrt{\frac{1}{N}\sum_{i=1}^{N}{\left(y_{i}-{\hat{y}}_{i}\right)}^{2}}$$

(6) $$\mathrm{NMSE}=\frac{\sum_{i=1}^{N}{\left(y_{i}-{\hat{y}}_{i}\right)}^{2}}{\sum_{i=1}^{N}{\left(y_{i}-\bar{y}\right)}^{2}}$$

Table 4 lists RMSE and NMSE under four different road surface conditions. The errors were evaluated from the driving data collected for the duration of 100 s. The results show that the orders of errors for NMSE range from 10^−3^ to 10^−1^ for the six vehicle states in the four road conditions. The vehicle state estimation module represents the nonlinear vehicle dynamics with high level of accuracy.

### 4.3. Detection of Driver Intention

The driver’s intention estimated by the trained SVM was compared with the driver’s true intention for lane change. The SVM was trained with six feature sets with six different combinations of the input signals (Table 2). Table 5 compares the accuracy rates with the six feature sets under four road surface conditions.

The results show that the feature set with the yaw rate, the longitudinal acceleration, the steering wheel angle, the lateral velocity, the roll rate and the heading direction (Set 6) shows the highest accuracy rate of detection in any road surface condition. It should be noted that the accuracy of detection is better than that of the feature set with all the available input signals (Set 4) and that of the feature set with on-board sensor measurements only (Set 1). Sets 4–6 with the heading direction show much higher accuracy rates than Sets 1–3 without the heading direction. Thus, the heading direction is an input signal of major importance for identifying the driver’s intention for lane change.

**Figure 16:**
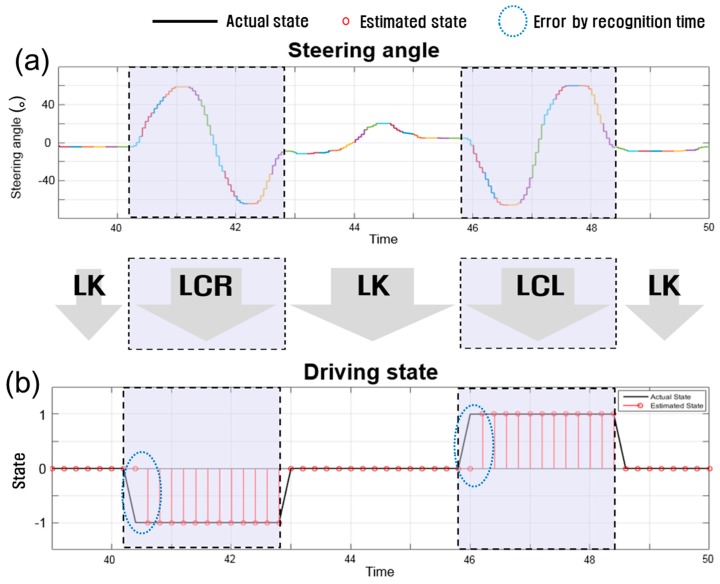
Lane change maneuvers and driver’s intention: (**a**) steering wheel angle; and (**b**) driving state (−1 is LCR, 0 is LK, 1 is LCL).

Figure 16 shows typical lane change maneuvers on dry asphalt and the driver’s intentions classified by the driver intention detection module using the optimal feature set (Set 6). Figure 16a plots the steering wheel angle during lane change maneuvers. Figure 16b compares the driver’s true intention (solid line) and the detected intention by the module (marked by o). In Figure 16, LCR, LK, and LCL are labeled as −1, 0, and 1, respectively.

As can be seen in Figure 16, the module correctly detected the driver’s intention for lane change, while there are time delays before correct detections. The delay is mainly attributed to the update rate of detection, which is dependent on the window slide size (0.2 s). The module requires a time longer than 0.2 s to determine the driver’s intention from sufficient information on the pattern of lane change maneuver. In addition, it appears that this time delay mainly contributes to the errors listed in Table 5.

Table 6 lists the average time to correctly detect the driver’s intention from the onset of the driver’s lane change maneuver under four different road surface conditions. The results show that the average time delays for LCL and LCR range from 0.4 to 0.45 s, while that for LK is between 0.146 and 0.222 s. The experimental results demonstrate that the trained driver intention detection module can identify the driver’s intention accurately and quickly.

## 5. Conclusions

In this study, we propose a novel method to classify the driver’s intention for lane change, based on measured and estimated information on the driver’s control inputs, the vehicle states, and the road condition. By using machine learning-based estimation techniques, the road surface condition and the extra vehicle states are augmented from the measured data obtained from the basic on-board sensors, which are commonly equipped on recent passenger vehicles.

For the classification of the driver’s intention, an SVM-based model is employed. As the inputs to the SVM model, the road surface condition and the extra vehicle states are estimated by ANN-based models that are trained a priori using the dynamics simulation data from a driving simulator. The augmented information estimated by the ANN models (the friction coefficient of the road surface, the lateral velocity, the side slip angle, the lateral tire force, the roll rate, the spring compression, and the heading direction) is essential to capture the dynamic situations of the driving vehicle.

The effectiveness of the proposed method was tested using driving simulation data. The results demonstrate that the driver’s intention for lane change can be detected more accurately using both measured and estimated data than using only measured data. The simulation results also show that the classification accuracy is the highest with the yaw rate, the lateral acceleration, the steering wheel angle, the lateral velocity, the roll rate and the heading direction as the inputs to the SVM module, rather than with all the available information from measurement and estimation as the inputs. Among the estimated vehicle states, the heading direction, the lateral velocity, and the roll rate appear to play an important role to improve the classification accuracy of the SVM model. The classification accuracy with the augmented information was higher than 90% in any road surface condition.

The proposed method can be utilized as a preprocessing algorithm for the ADAS by accurately and effectively detecting the driver’s intention for lane change. The developed algorithm can allow us to replace expensive sensors with economical on-board sensor algorithms. Due to its superior computational efficiency to numerical integration, the ANN-based vehicle dynamics model can be more effective than complex differential equation-based approaches. The proposed method can also be applicable to analysis of the driver's driving pattern. Based on the analysis, the ADAS may be adaptively activated according to the driver’s driving pattern. 

For our future works, we are planning to implement the developed algorithm on actual vehicle systems with multiple human subjects with different characteristics. In addition, we will investigate other advanced machine learning algorithms, such as long short-term memory (LSTM), for different types of driver intentions. Another important issue with driving safety is human factors. It was reported that about 90% of vehicle accidents were caused by human errors [39]. We also plan to study algorithms to differentiate the driver’s true intention from the driver’s erroneous maneuver. 

## Figures and Tables

**Table 1:** Road friction coefficient and road surface condition.

Road Surface Conditions	Friction Coefficients
Dry Asphalt	0.8
Gravel	0.6
Wet	0.4
Snowy	0.3

**Table 2:** Combinations of Input Signals.

Feature Set	Combinations of Input Signals
1	Yaw rate, Longitudinal acceleration, Lateral acceleration, Steering wheel angle, Wheel speed
2	Yaw rate, Longitudinal acceleration, Lateral acceleration, Steering wheel angle, Wheel speed, **Lateral velocity, Roll rate**
3	Yaw rate, Longitudinal acceleration, Lateral acceleration, Steering wheel angle, Wheel speed, **sideslip angle, Lateral tire force, Spring compression**
4	Yaw rate, Longitudinal acceleration, Lateral acceleration, Steering wheel angle, Wheel speed, **Lateral velocity, Roll rate, sideslip angle, Lateral tire force, Spring compression, Heading**
5	Yaw rate, Lateral acceleration, Steering wheel angle, **Lateral velocity, Roll rate, sideslip angle, Lateral tire force, Spring compression, Heading**
6	Yaw rate, Lateral acceleration, Steering wheel angle, **Lateral velocity, Roll rate, Heading**

**Table 3:** Result of classification test depending on friction coefficient.

	1	2	3	4	5	6	7	8	9	10	11	12	13	Rate
Dry Asphalt (NS)	NS 87.9%	NS 69.6%	NS 79.8%	NS 75.9%	NS 96.0%	NS 83.2%	NS 57.9%	NS 77.5%	NS 94.2%	NS 77.1%	NS 63.9%	NS 96.7%	NS 96.5%	13/13
Gravel (NS)	NS 92.5%	NS 70.5%	NS 93.0%	NS 71.0%	NS 67.0%	NS 53.3%	NS 53.6%	NS 75.7%	NS 88.4%	NS 75.9%	NS 79.9%	NS 94.7%	NS 100%	13/13
Wet (S)	S 58.1%	S 93.4%	S 91.5%	S 53.6%	S 82.3%	S 100%	S 87.8%	S 100%	S 92.9%	S 95.6%	S 96.2%	S 95.7%	S 65.4%	13/13
Snowy (S)	S 100%	S 62.7%	S 100%	S 76.0%	S 80.8%	S 100%	S 72.8%	NS 56.1%	S 73.4%	S 77.0%	S 95.0%	S 100%	S 100%	12/13

**Table 4:** Range of data, RMSE and NMSE in each case.

	Road Condition	Data	RMSE	NMSE	Order (NMSE)
Lateral Velocity	Dry asphalt	−0.6~0.6	0.0148	0.0139	10^−2^
	Gravel	−0.6~0.6	0.0229	0.0195	
	Wet	−0.6~0.6	0.0218	0.0082	
	Snowy	−0.6~0.6	0.0236	0.0077	
Side Slip Angle	Dry asphalt	−0.6~0.6	0.0133	0.0123	10^−2^
	Gravel	−0.6~0.6	0.0233	0.0167	
	Wet	−0.9~0.9	0.0374	0.0142	
	Snowy	−0.9~0.9	0.0403	0.0097	
Lateral Tire Force	Dry asphalt	−3000~2000	34.6	0.00089	10^−3^
	Gravel	−3000~2000	84.6	0.0066	
	Wet	−2500~2000	38.2	0.0021	
	Snowy	−2000~2000	27.5	0.0021	
Roll rate	Dry asphalt	−8~8	0.332	0.0304	10^−1^
	Gravel	−6~6	0.363	0.0475	
	Wet	−5~5	0.353	0.0774	
	Snowy	−4~4	0.304	0.1096	
Spring Compression	Dry asphalt	50~85	0.708	0.0145	10^−2^
	Gravel	50~85	0.962	0.0303	
	Wet	60~80	0.594	0.0192	
	Snowy	60~80	0.698	0.0479	
Heading	Dry asphalt	−15~15	1.05	0.0226	10^−2^
	Gravel	−15~15	0.962	0.0154	
	Wet	−15~15	0.551	0.008	
	Snowy	−15~15	0.545	0.0166	

**Table 5:** Detection accuracy in four different road conditions.

	Set 1 (%)	Set 2 (%)	Set 3 (%)	Set 4 (%)	Set 5 (%)	Set 6 (%)
(a) Dry Asphalt						
LCL	70.51	71.79	65.38	88.46	91.03	91.03
LK	96.30	95.06	95.68	96.91	96.91	96.91
LCR	67.14	74.29	75.71	91.43	90.00	91.43
(b) Gravel						
LCL	66.15	72.31	56.92	90.77	92.30	92.30
LK	95.57	96.20	95.57	96.20	96.84	96.84
LCR	56.96	68.35	64.56	89.87	89.87	91.14
(c) Wet						
LCL	54.29	67.14	60.00	92.86	92.86	92.86
LK	97.14	97.71	97.71	97.71	97.71	97.14
LCR	60.66	73.77	70.49	90.16	90.16	90.16
(d) Snowy						
LCL	62.26	62.26	52.83	90.57	90.57	90.57
LK	97.84	97.84	97.84	97.30	97.30	97.30
LCR	71.43	73.21	75.00	89.29	89.29	91.07

**Table 6:** Average time delay for correct detection.

Driver Maneuver	Dry Asphalt	Gravel	Wet	Snowy
LCL	0.45 s	0.4 s	0.4 s	0.4 s
LK	0.15 s	0.222 s	0.182 s	0.146 s
LCR	0.45 s	0.433 s	0.433 s	0.4 s
